# Supplementary material for: Consumption in the G20 nations causes particulate air pollution resulting in two million premature deaths annually
Source: Nat Commun. 2021 Nov 2;12:6286. doi: 10.1038/s41467-021-26348-y (PMC8563796; doi:10.1038/s41467-021-26348-y)
Supplement: Supplementary file 1 — Supplementary Information [file 41467_2021_26348_MOESM1_ESM.pdf]

Supporting Information

Consumption in the G20 nations causes particulate air pollution resulting in two million premature deaths annually

By

Keisuke Nansai*, Susumu Tohno, Satoru Chatani, Keiichiro Kanemoto,

Shigemi Kagawa, Yasushi Kondo, Wataru Takayanagi, and Manfred Lenzen

DOI: 10.1038/s41467-021-26348-y

Contents

Supplementary Tables 1–3

Supplementary Figures 1–20

Integrated exposure-response (IER) model

* Corresponding author

Material Cycles Division, National Institute for Environmental Studies, 16-2 Onogawa, Tsukuba, Ibaraki 305-8506, Japan

Tel.: +81 29-850-2889, E-mail: nansai.keisuke@nies.go.jp

Supplementary Table 1: Country and region codes used in this study

No.	Code	Name	No.	Code	Name	No.	Code	Name	No.	Code	Name
1	ABW	Aruba	51	DOM	Dominican Republic	101	LBN	Lebanon	151	QAT	Qatar
2	AFG	Afghanistan	52	DZA	Algeria	102	LBR	Liberia	152	ROU	Romania
3	AGO	Angola	53	ECU	Ecuador	103	LBY	Libya	153	RUS	Russian Federation
4	ALB	Albania	54	EGY	Egypt, Arab Rep.	104	LCA	St. Lucia	154	RWA	Rwanda
5	AND	Andorra	55	ERI	Eritrea	105	LIE	Liechtenstein	155	SAU	Saudi Arabia
6	ARE	United Arab Emirates	56	ESP	Spain	106	LKA	Sri Lanka	156	SDN	Sudan
7	ARG	Argentina	57	EST	Estonia	107	LSO	Lesotho	157	SEN	Senegal
8	ARM	Armenia	58	ETH	Ethiopia	108	LTU	Lithuania	158	SGP	Singapore
9	AUS	Australia	59	FIN	Finland	109	LUX	Luxembourg	159	SLB	Solomon Islands
10	AUT	Austria	60	FRA	France	110	LVA	Latvia	160	SLE	Sierra Leone
11	AZE	Azerbaijan	61	FRO	Faroe Islands	111	MAC	Macao SAR, China	161	SLV	El Salvador
12	BDI	Burundi	62	GAB	Gabon	112	MAR	Morocco	162	SMR	San Marino
13	BEL	Belgium	63	GBR	United Kingdom	113	MCO	Monaco	163	SOM	Somalia
14	BEN	Benin	64	GEO	Georgia	114	MDA	Moldova	164	SRB	Serbia
15	BFA	Burkina Faso	65	GHA	Ghana	115	MDG	Madagascar	165	SSD	South Sudan
16	BGD	Bangladesh	66	GIB	Gibraltar	116	MDV	Maldives	166	STP	Sao Tome and Principe
17	BGR	Bulgaria	67	GIN	Guinea	117	MEX	Mexico	167	SUR	Suriname
18	BHR	Bahrain	68	GMB	Gambia	118	MKD	Macedonia, FYR	168	SVK	Slovak Republic
19	BHS	Bahamas	69	GNB	Guinea-Bissau	119	MLI	Mali	169	SVN	Slovenia
20	BIH	Bosnia and Herzegovina	70	GNQ	Equatorial Guinea	120	MLT	Malta	170	SWE	Sweden
21	BLR	Belarus	71	GRC	Greece	121	MMR	Myanmar	171	SWZ	Swaziland
22	BLZ	Belize	72	GRD	Grenada	122	MNE	Montenegro	172	SYC	Seychelles
23	BMU	Bermuda	73	GRL	Greenland	123	MNG	Mongolia	173	SYR	Syrian Arab Republic
24	BOL	Bolivia	74	GTM	Guatemala	124	MNP	Northern Mariana Islands	174	TCA	Turks and Caicos Islands
25	BRA	Brazil	75	GUM	Guam	125	MOZ	Mozambique	175	TCD	Chad
26	BRB	Barbados	76	GUY	Guyana	126	MRT	Mauritania	176	TGO	Togo
27	BRN	Brunei Darussalam	77	HKG	Hong Kong SAR, China	127	MWI	Malawi	177	THA	Thailand
28	BTN	Bhutan	78	HND	Honduras	128	MYS	Malaysia	178	TJK	Tajikistan
29	BWA	Botswana	79	HRV	Croatia	129	NAM	Namibia	179	TKM	Turkmenistan
30	CAF	Central African Republic	80	HTI	Haiti	130	NCL	New Caledonia	180	TLS	Timor-Leste
31	CAN	Canada	81	HUN	Hungary	131	NER	Niger	181	TTO	Trinidad and Tobago
32	CHE	Switzerland	82	IDN	Indonesia	132	NGA	Nigeria	182	TUN	Tunisia
33	CHI	Channel Islands	83	IMN	Isle of Man	133	NIC	Nicaragua	183	TUR	Turkey
34	CHL	Chile	84	IND	India	134	NLD	Netherlands	184	TWN	Taiwan, China
35	CHN	China	85	IRL	Ireland	135	NOR	Norway	185	TZA	Tanzania
36	CIV	Cote d'Ivoire	86	IRN	Iran, Islamic Rep.	136	NPL	Nepal	186	UGA	Uganda
37	CMR	Cameroon	87	IRQ	Iraq	137	NRU	Nauru	187	UKR	Ukraine
38	COD	Congo, Dem. Rep.	88	ISL	Iceland	138	NZL	New Zealand	188	URY	Uruguay
39	COG	Congo, Rep.	89	ISR	Israel	139	OMN	Oman	189	USA	United States
40	COL	Colombia	90	ITA	Italy	140	PAK	Pakistan	190	UZB	Uzbekistan
41	COM	Comoros	91	JAM	Jamaica	141	PAN	Panama	191	VCT	St. Vincent and the Grenadines
42	CRI	Costa Rica	92	JOR	Jordan	142	PER	Peru	192	VEN	Venezuela, RB
43	CUB	Cuba	93	JPN	Japan	143	PHL	Philippines	193	VNM	Vietnam
44	CUW	Curacao	94	KAZ	Kazakhstan	144	PLW	Palau	194	VUT	Vanuatu
45	CYM	Cayman Islands	95	KEN	Kenya	145	PNG	Papua New Guinea	195	XKX	Kosovo
46	CYP	Cyprus	96	KGZ	Kyrgyz Republic	146	POL	Poland	196	YEM	Yemen, Rep.
47	CZE	Czech Republic	97	KHM	Cambodia	147	PRK	Korea, Dem. People's Rep.	197	ZAF	South Africa
48	DEU	Germany	98	KOR	Korea, Rep.	148	PRT	Portugal	198	ZMB	Zambia
49	DJI	Djibouti	99	KWT	Kuwait	149	PRY	Paraguay	199	ZWE	Zimbabwe
50	DNK	Denmark	100	LAO	Lao PDR	150	PSE	West Bank and Gaza			

Supplementary Table 2: Parameters of the integrated exposure–response functions

End point			Parameters of IER functions			
(Disease)	Age (y)	Range	α	γ	δ	C_0
LRI	All ages	0.025	1.3969	0.0133	0.8529	5
		0.5	1.5975	0.0119	0.8785	4.2
		0.975	1.7704	0.0097	0.9298	3.3
COPD	All ages	0.025	4.5814	0.0068	0.6341	5
		0.5	5.0893	0.0082	0.6128	4.2
		0.975	5.6845	0.0088	0.6132	3.3
Lung cancer	All ages	0.025	19.0060	0.0006	0.8140	5
		0.5	21.3111	0.0007	0.7854	4.2
		0.975	23.9074	0.0008	0.7607	3.3
Stroke	25–29	0.025	2.5926	0.0203	0.5267	5
		0.5	3.1663	0.0230	0.5036	4.2
		0.975	3.9343	0.0234	0.4933	3.3
	30–34	0.025	1.9052	0.0232	0.5526	5
		0.5	2.6145	0.0243	0.5146	4.2
		0.975	3.5085	0.0248	0.4873	3.3
	35–39	0.025	1.9076	0.0236	0.5341	5
		0.5	2.1195	0.0283	0.5188	4.2
		0.975	4.5801	0.0183	0.4710	3.3
	40–44	0.025	1.4416	0.0276	0.5451	5
		0.5	2.3848	0.0259	0.4822	4.2
		0.975	3.0749	0.0259	0.4659	3.3
	45–49	0.025	1.7349	0.0237	0.5067	5
		0.5	2.1095	0.0270	0.4808	4.2
		0.975	2.2997	0.0334	0.4639	3.3
	50–54	0.025	1.4240	0.0249	0.5183	5
		0.5	1.7503	0.0303	0.4745	4.2
		0.975	1.9634	0.0331	0.4761	3.3
	55–59	0.025	1.0336	0.0325	0.5252	5
		0.5	1.7046	0.0317	0.4499	4.2
		0.975	1.9530	0.0343	0.4455	3.3
	60–64	0.025	1.0537	0.0248	0.5353	5
		0.5	1.1653	0.0370	0.4785	4.2
		0.975	1.5890	0.0358	0.4557	3.3
	65–69	0.025	0.7889	0.0365	0.5044	5
		0.5	1.0896	0.0374	0.4663	4.2
		0.975	1.5057	0.0366	0.4339	3.3
	70–74	0.025	0.6920	0.0401	0.4886	5
		0.5	0.9758	0.0414	0.4380	4.2
		0.975	1.0577	0.0477	0.4331	3.3
	75–79	0.025	0.5782	0.0401	0.4967	5
		0.5	0.8914	0.0386	0.4369	4.2
		0.975	1.0480	0.0448	0.4096	3.3
	80–	0.025	0.4761	0.0417	0.4913	5
		0.5	0.6047	0.0489	0.4444	4.2
		0.975	0.8996	0.0466	0.3959	3.3

Supplementary Table 2: Parameters of the integrated exposure–response functions (cont.)

End point (Disease)	Age (y)	Range	Parameters of IER functions α	γ	δ	C_0
IHD	25–29	0.025	5.6431	0.0189	0.4171	5
		0.5	6.4134	0.0226	0.3897	4.2
		0.975	7.2581	0.0287	0.3570	3.3
	30–34	0.025	4.9182	0.0197	0.4156	5
		0.5	5.6899	0.0247	0.3779	4.2
		0.975	6.2127	0.0297	0.3584	3.3
	35–39	0.025	3.7269	0.0244	0.4157	5
		0.5	4.1968	0.0316	0.3752	4.2
		0.975	4.6216	0.0398	0.3451	3.3
	40–44	0.025	3.4398	0.0248	0.4102	5
		0.5	3.6596	0.0343	0.3685	4.2
		0.975	3.3558	0.0492	0.3542	3.3
	45–49	0.025	3.3969	0.0237	0.3948	5
		0.5	3.8844	0.0304	0.3538	4.2
		0.975	4.2263	0.0366	0.3353	3.3
	50–54	0.025	3.0070	0.0251	0.3870	5
		0.5	3.1993	0.0321	0.3606	4.2
		0.975	3.2575	0.0424	0.3357	3.3
	55–59	0.025	3.0724	0.0226	0.3786	5
		0.5	3.4114	0.0288	0.3437	4.2
		0.975	3.7147	0.0348	0.3212	3.3
	60–64	0.025	3.5732	0.0188	0.3571	5
		0.5	4.0402	0.0226	0.3282	4.2
		0.975	4.1537	0.0298	0.3009	3.3
	65–69	0.025	2.0366	0.0294	0.3644	5
		0.5	2.2770	0.0365	0.3307	4.2
		0.975	2.4855	0.0436	0.3062	3.3
	70–74	0.025	1.4329	0.0365	0.3665	5
		0.5	1.5980	0.0474	0.3280	4.2
		0.975	1.6957	0.0580	0.3054	3.3
	75–79	0.025	1.1951	0.0384	0.3567	5
		0.5	1.2501	0.0504	0.3291	4.2
		0.975	1.3643	0.0615	0.3029	3.3
	80–	0.025	1.1401	0.0376	0.3394	5
		0.5	1.2915	0.0432	0.3139	4.2
		0.975	1.3342	0.0557	0.2882	3.3

Supplementary Table 3: Data for estimating $PM_{2.5}$ premature deaths per capita lifetime consumption

G20 nations	Per capita $PM_{2.5}$ premature death footprint in 2010	95% CI	$PM_{2.5}$ premature death per capita lifetime consumption	95% CI	Population	Life expectancy
	[Deaths x 10^{-3}/cap]	[Deaths x 10^{-3}/cap]	[Deaths /cap]	[Deaths /cap]	[1000 persons]	[years]
Argentina	0.14	[0.11 , 0.16]	0.011	[0.009 , 0.013]	40,896	76.52
Australia	0.26	[0.21 , 0.31]	0.021	[0.017 , 0.026]	22,155	82.75
Brazil	0.15	[0.12 , 0.19]	0.012	[0.009 , 0.014]	195,714	75.67
Canada[#]	0.40	[0.32 , 0.48]	0.032	[0.026 , 0.039]	34,148	81.95
China	0.66	[0.57 , 0.75]	0.051	[0.044 , 0.058]	1,368,811	76.70
France[#]	0.44	[0.36 , 0.53]	0.037	[0.030 , 0.044]	62,880	82.53
Germany[#]	0.54	[0.45 , 0.65]	0.044	[0.036 , 0.052]	80,827	80.99
India	0.40	[0.35 , 0.45]	0.028	[0.024 , 0.031]	1,234,281	69.42
Indonesia	0.22	[0.17 , 0.26]	0.016	[0.012 , 0.019]	241,834	71.51
Italy[#]	0.38	[0.31 , 0.45]	0.031	[0.026 , 0.037]	59,325	82.95
Japan[#]	0.33	[0.27 , 0.38]	0.027	[0.023 , 0.032]	128,542	84.21
Mexico	0.13	[0.11 , 0.15]	0.010	[0.008 , 0.012]	114,093	74.99
Russia	0.52	[0.42 , 0.62]	0.038	[0.031 , 0.045]	143,479	72.66
Saudi Arabia	0.45	[0.39 , 0.51]	0.034	[0.029 , 0.038]	27,421	75.00
South Africa	0.39	[0.33 , 0.45]	0.025	[0.021 , 0.029]	51,217	63.86
South Korea	0.41	[0.34 , 0.47]	0.034	[0.028 , 0.039]	49,546	82.63
Turkey	0.36	[0.30 , 0.42]	0.028	[0.023 , 0.032]	72,327	77.44
United Kingdom[#]	0.54	[0.44 , 0.64]	0.044	[0.036 , 0.052]	63,460	81.36
United States[#]	0.45	[0.36 , 0.54]	0.035	[0.029 , 0.043]	309,011	78.54
G7 total	0.44	[0.36 , 0.52]	0.036	[0.029 , 0.043]	738,193	81.79
G20 total	0.46	[0.39 , 0.53]	0.036	[0.030 , 0.041]	4,299,966	77.46

#) G7 nations

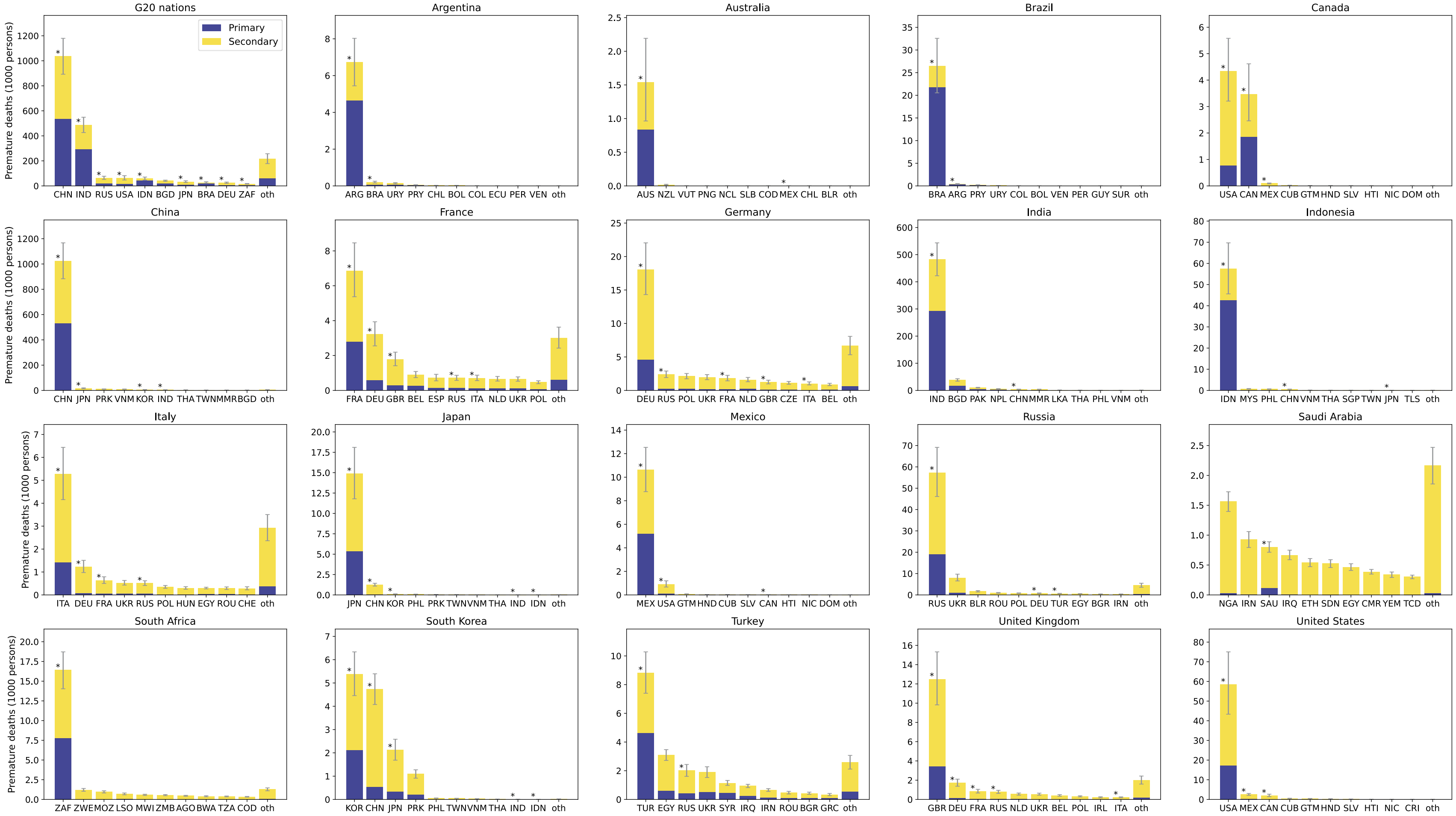

Supplementary Figure 1: Top 10 countries with most premature deaths associated with production-based $PM_{2.5}$ emissions of each G20 nation in 2010; Asterisks above bars indicate G20 nations with presidency rights. Country and region codes within the top 10 are as listed in Supplementary Table 1; others are aggregated as ‘oth’. Error bars indicate 95% confidence intervals. The centre of the bars indicates the mean value.

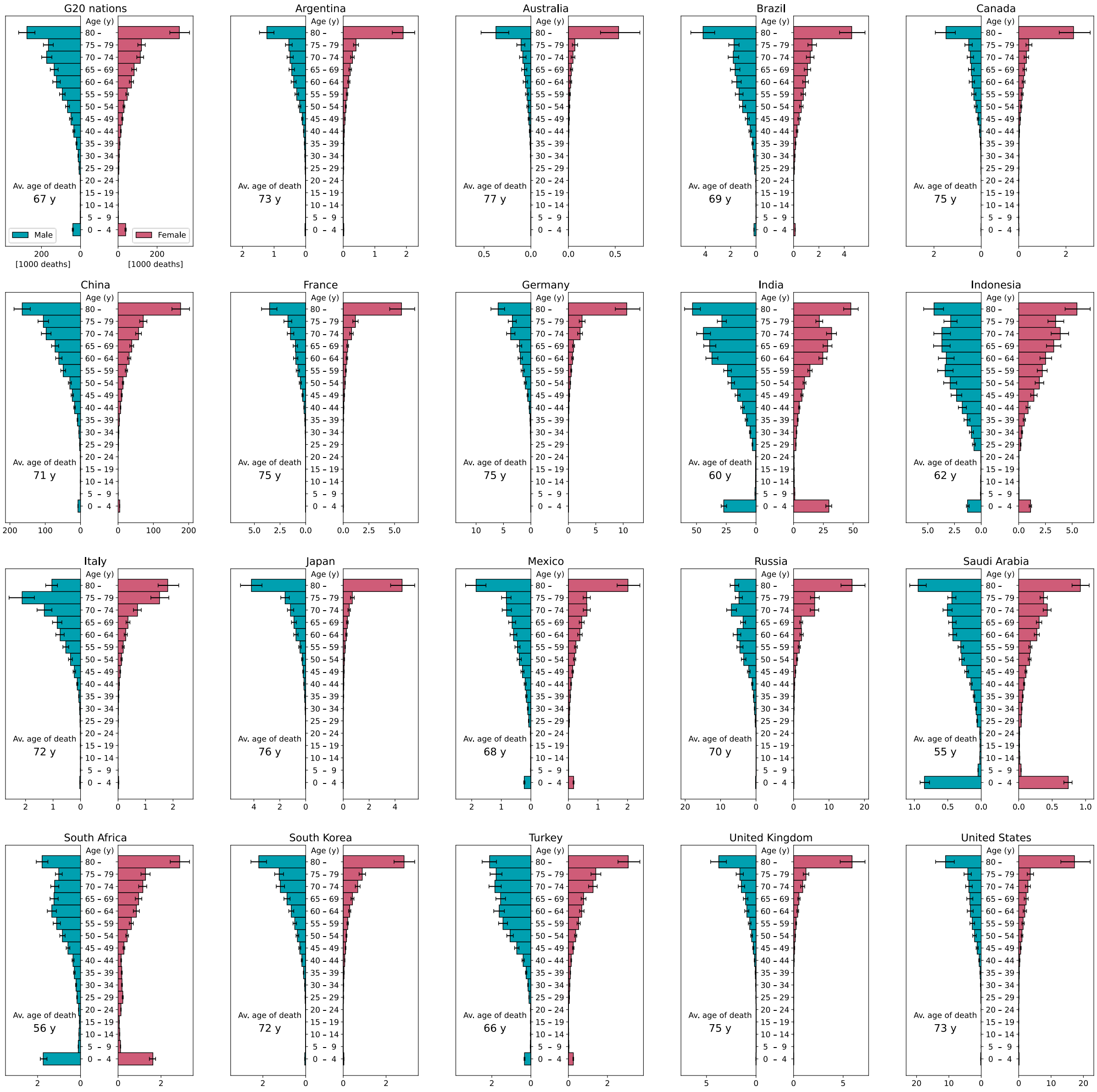

Supplementary Figure 2: Age and gender breakdown of premature deaths associated with production-based $PM_{2.5}$ emissions of each G20 nation in 2010; Error bars indicate 95% confidence intervals. The centre of the bars indicates the mean value.

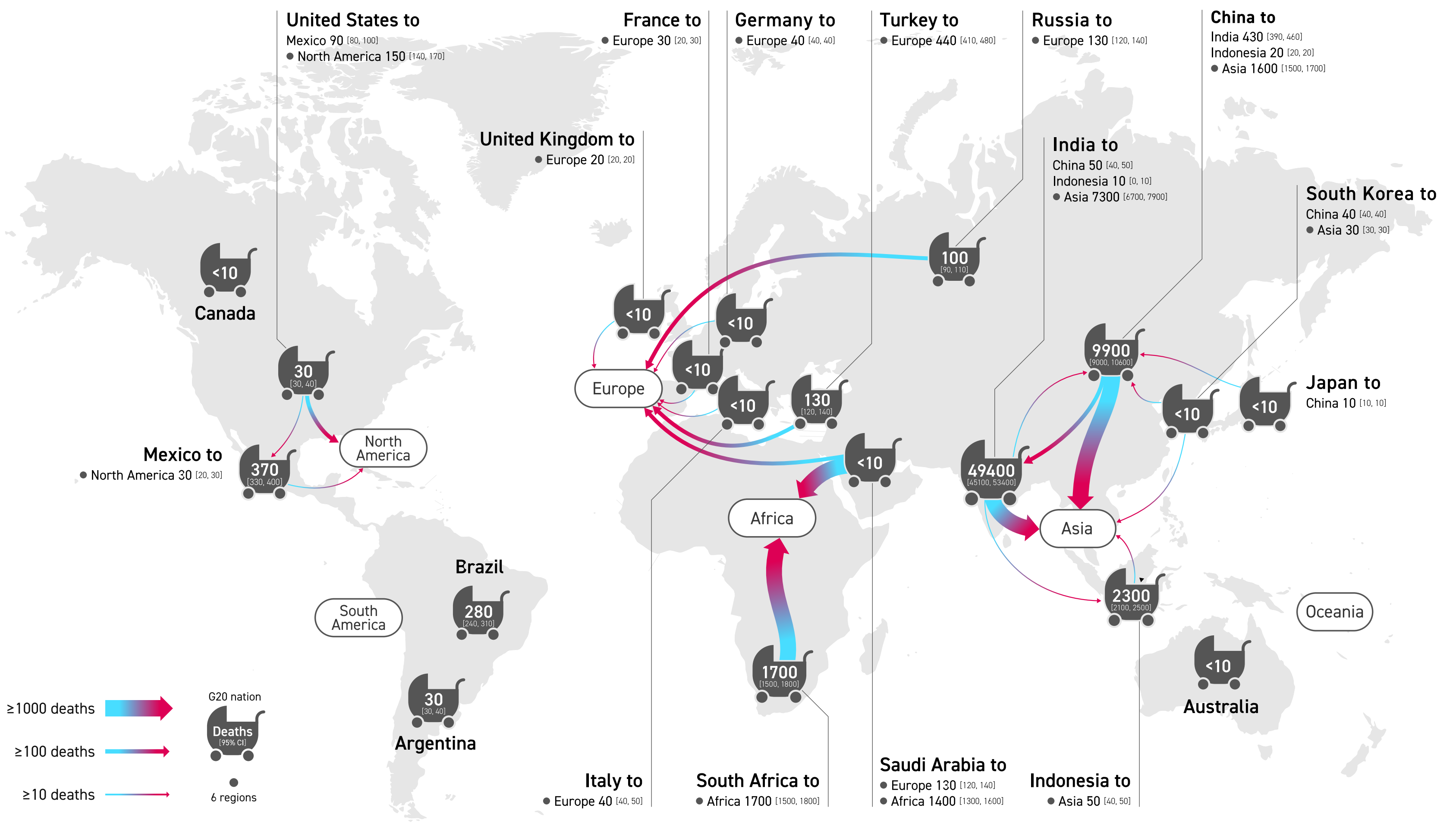

Supplementary Figure 3: Relationships between emission countries and impacted countries ("to") for infant deaths (zero to less than five years old) in $PM_{2.5}$ premature death caused by production-based emissions of G20 nations

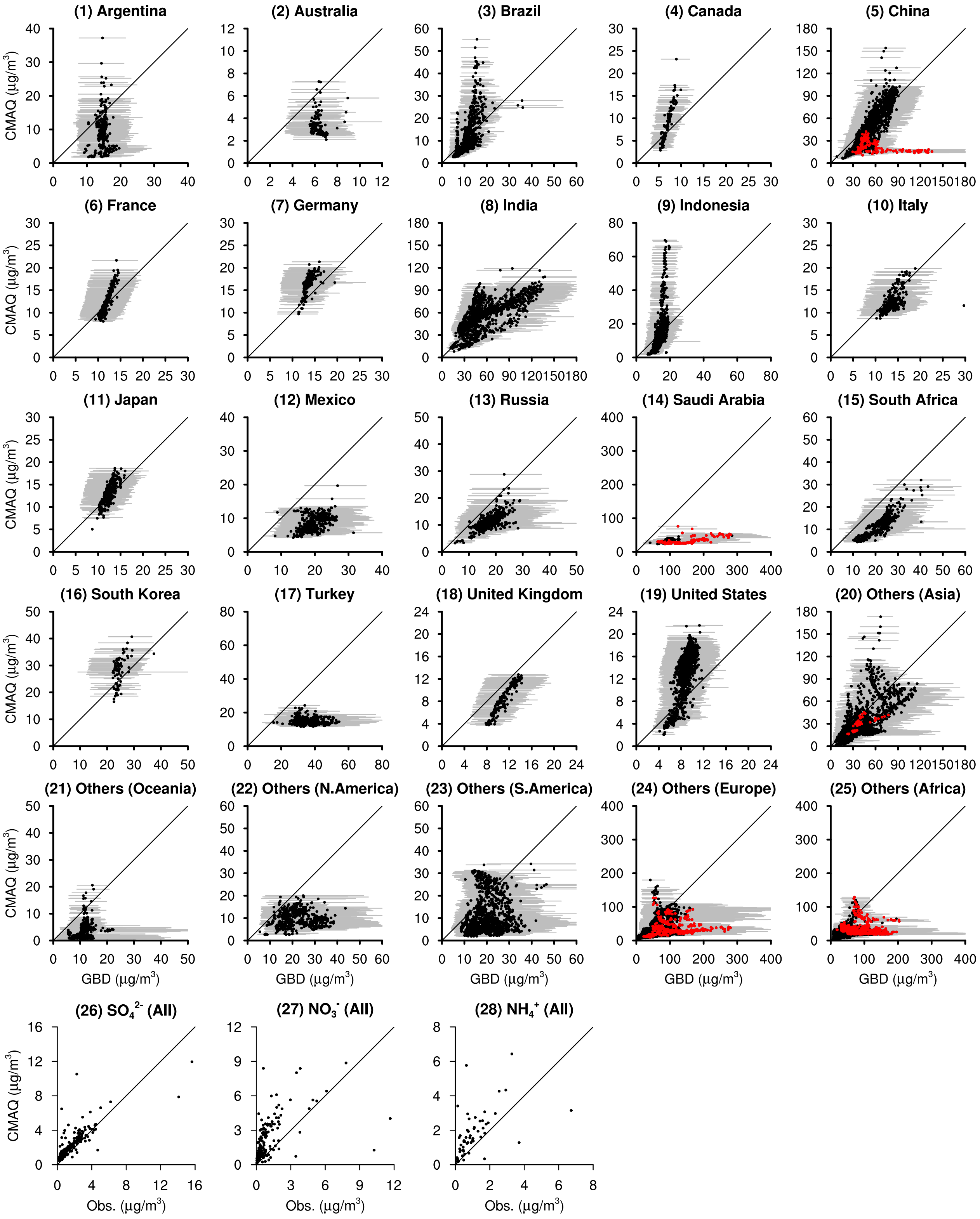

Supplementary Figure 4: Comparison of CMAQ-simulated $PM_{2.5}$ concentrations using base-case emission map with GBD2016 concentration data (black dots, Figures (1)-(25)) with

the 95% CI range (grey lines); red dots are grid squares with dust concentration indicated in GBD2013; grid squares with a population density over 22.5 persons/km^2 are shown.

Comparison of CMAQ-simulated $PM_{2.5}$ secondary particle concentrations with observation data in IMPROVE (http://vista.cira.colostate.edu/improve/Data/data.htm), EMEP (https://projects.nilu.no/ccc/index.html) and EANET (https://monitoring.eanet.asia/document/public/index), respectively (Figures (26)-(28))

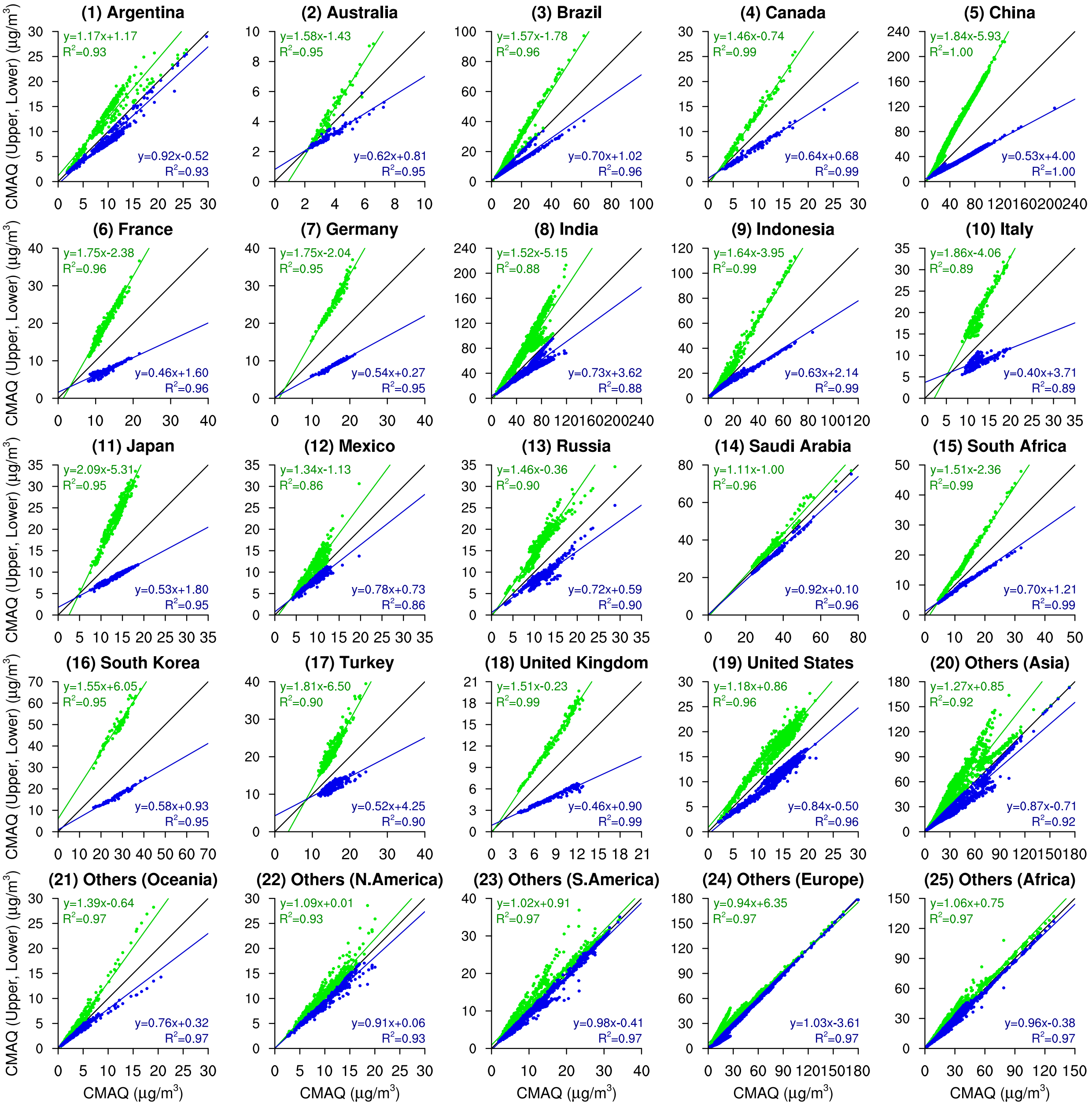

Supplementary Figure 5: Comparison of CMAQ-simulated $PM_{2.5}$ concentrations with mean emissions of EDGAR using upper and lower bound emissions in 95% CI of EDGAR (green: with upper bound emissions, blue: with lower bound emissions)

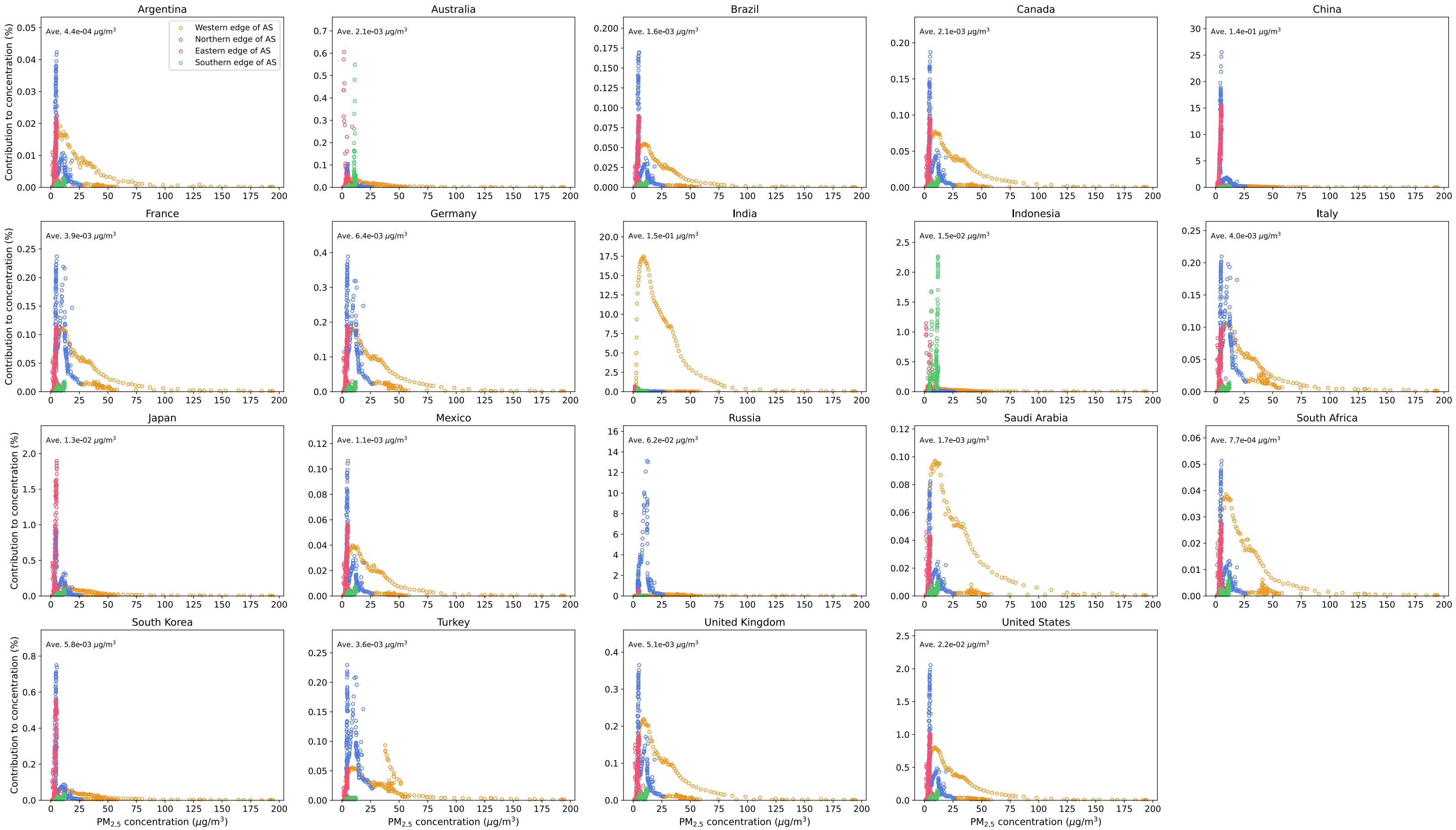

Supplementary Figure 6: CMAQ-simulated $PM_{2.5}$ concentration in grid squares on western, northern, eastern and southern edges of Asian (AS) region and contribution of consumption-based emissions of each G20 nation to $PM_{2.5}$ concentration in those squares

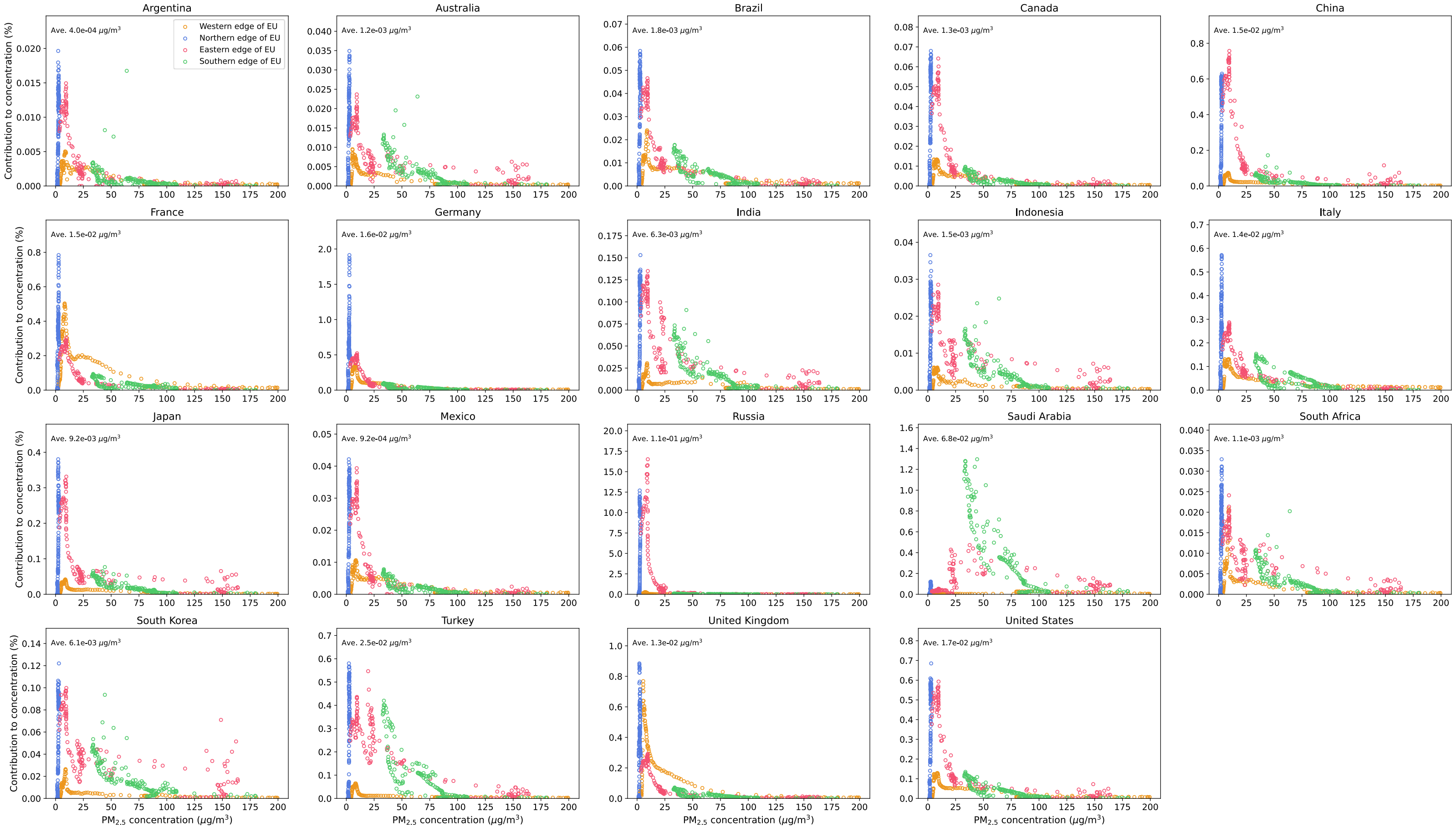

Supplementary Figure 7: CMAQ-simulated $PM_{2.5}$ concentration in grid squares on western, northern, eastern and southern edges of European (EU) region and contribution of consumption-based emissions of each G20 nation to $PM_{2.5}$ concentration in those squares

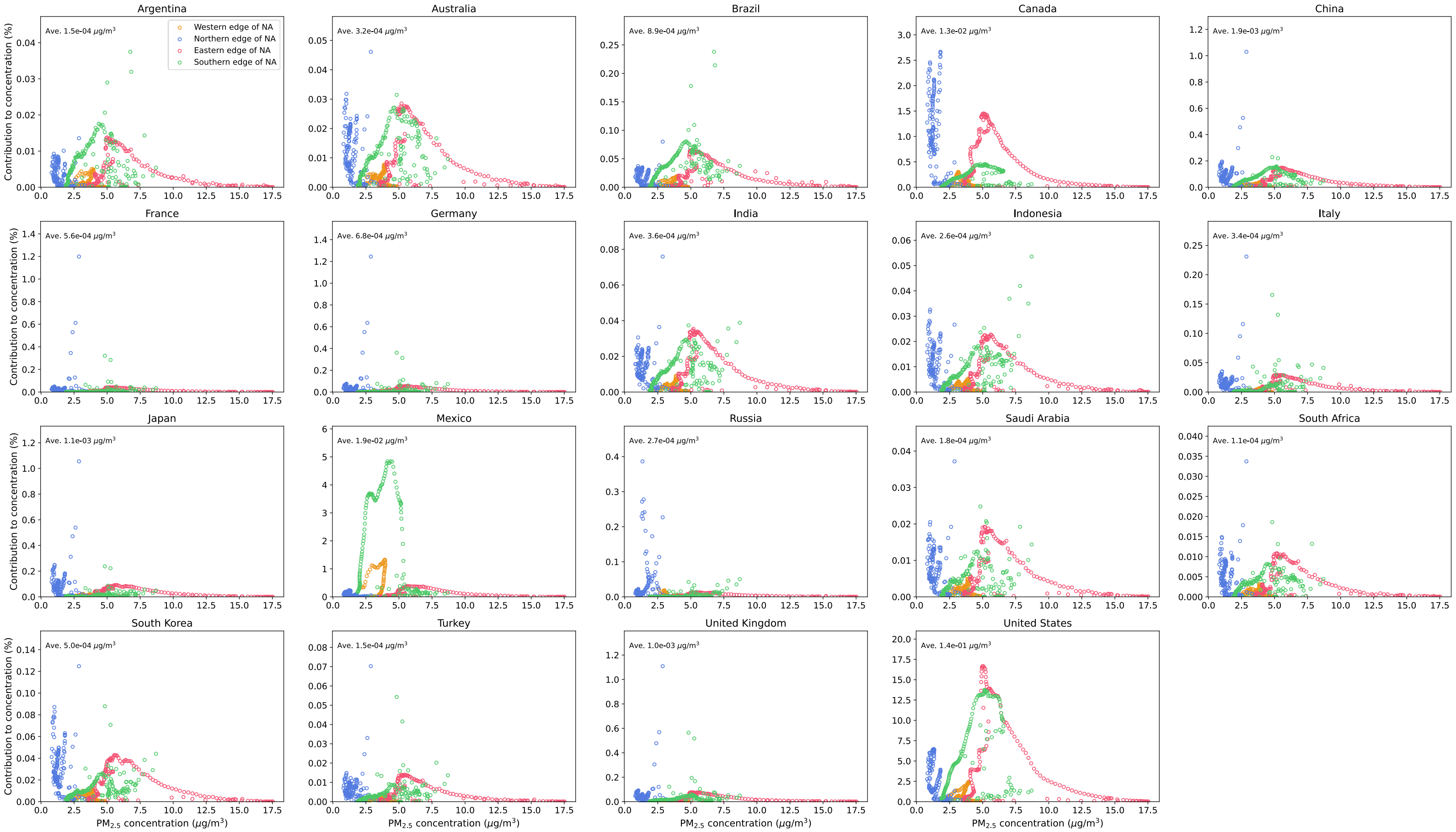

Supplementary Figure 8: CMAQ-simulated $PM_{2.5}$ concentration in grid squares on western, northern, eastern and southern edges of North American (NA) region and contribution of consumption-based emissions of each G20 nation to $PM_{2.5}$ concentration in those squares

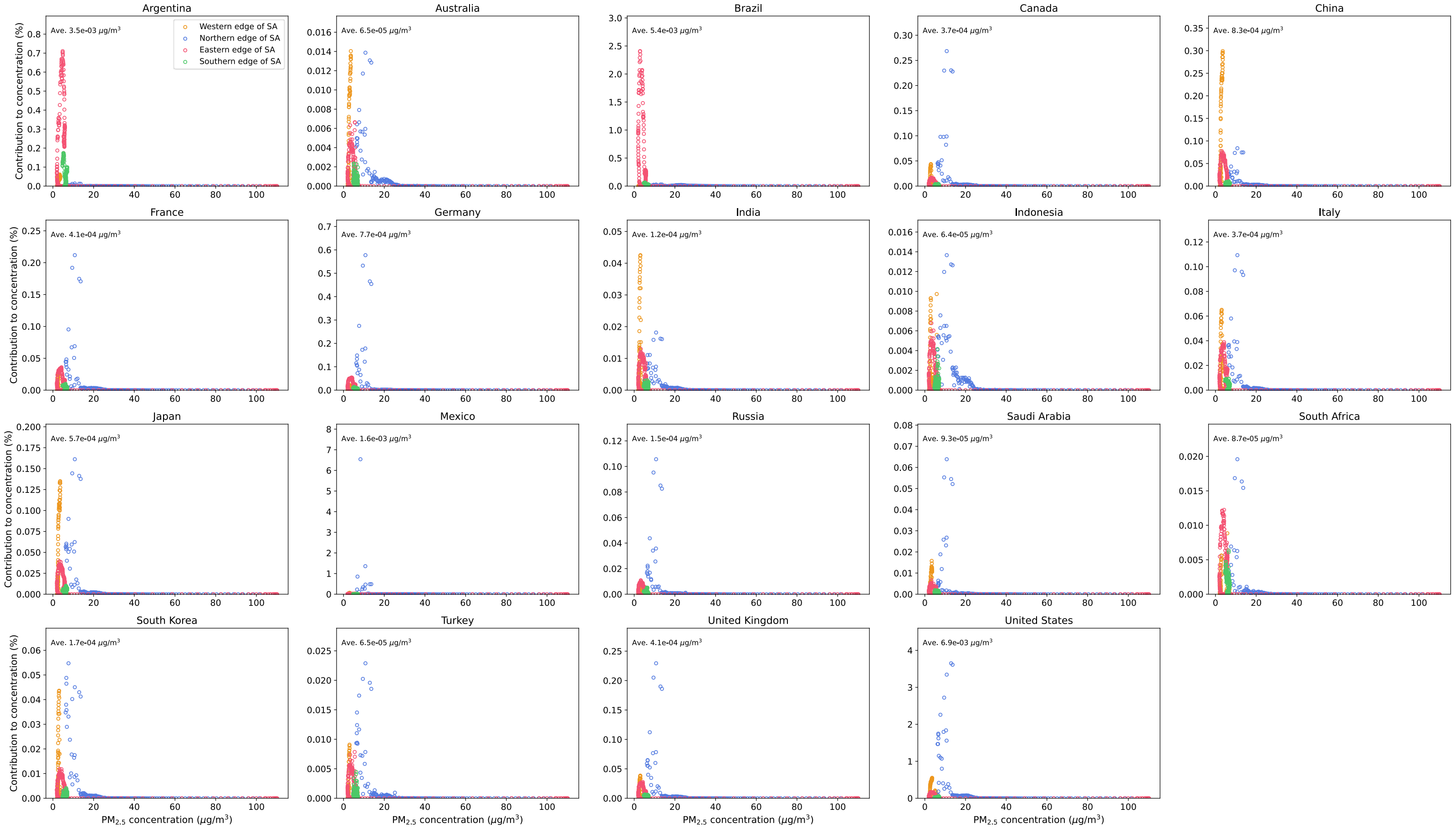

Supplementary Figure 9: CMAQ-simulated $PM_{2.5}$ concentration in grid squares at western, northern, eastern and southern edges of South American (SA) region and contribution of consumption-based emissions of each G20 nation to $PM_{2.5}$ concentration in those squares

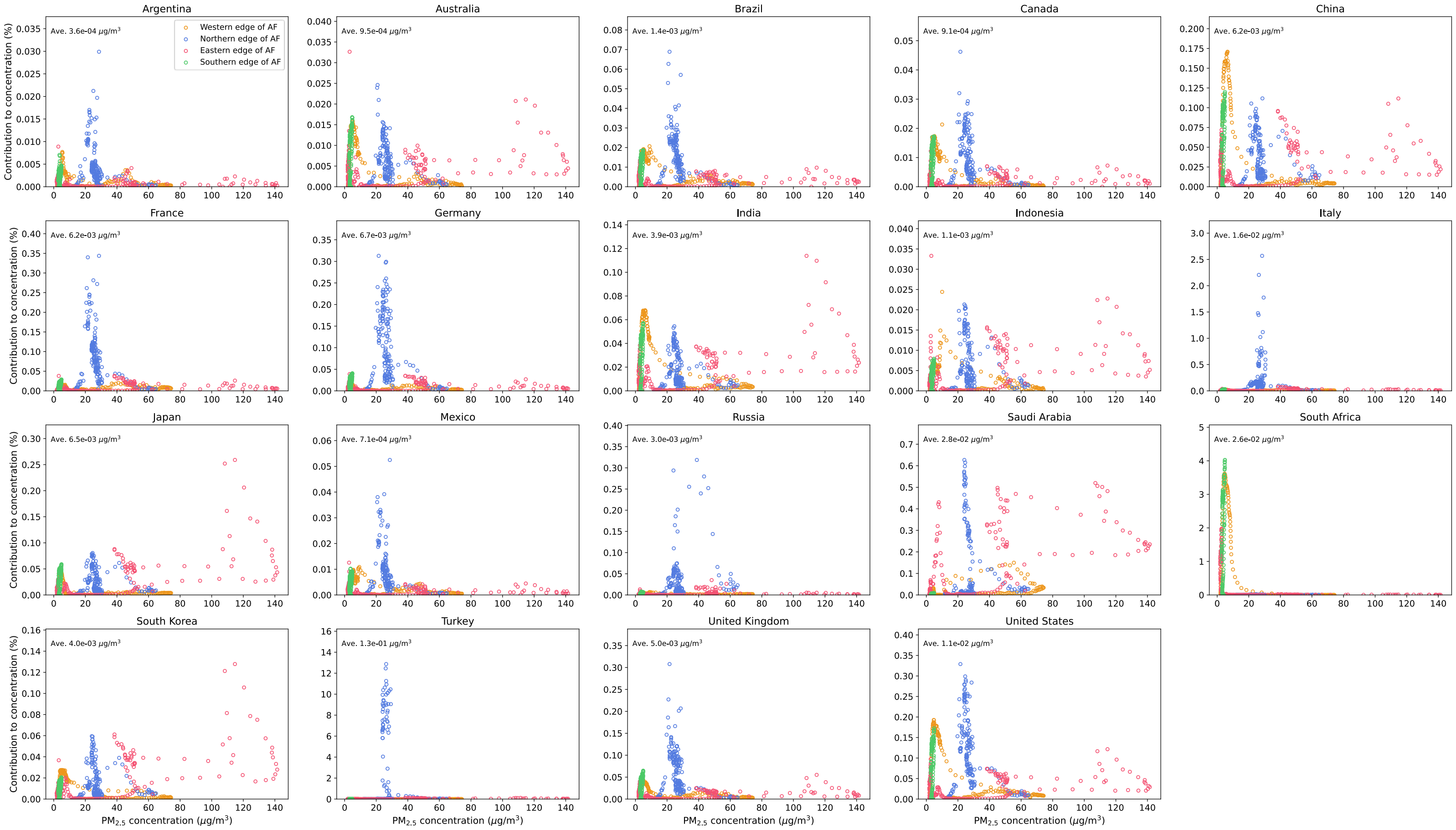

Supplementary Figure 10: CMAQ-simulated $PM_{2.5}$ concentration in grid squares on western, northern, eastern and southern edges of African (AF) region and contribution of consumption-based emissions of each G20 nation to $PM_{2.5}$ concentration in those squares

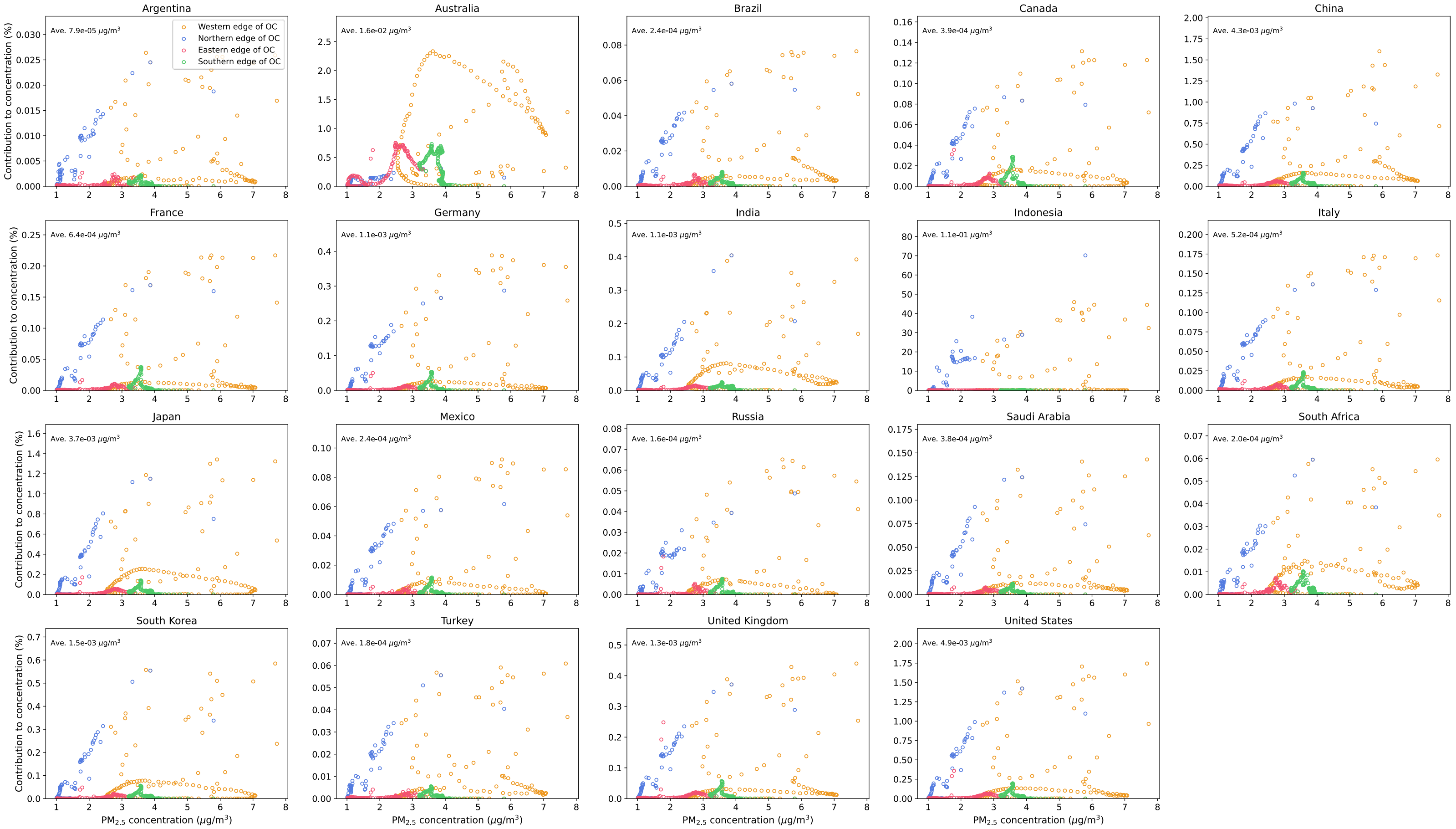

Supplementary Figure 11: CMAQ-simulated $PM_{2.5}$ concentration in grid squares at western, northern, eastern and southern edges of Oceania (OS) region and contribution of consumption-based emissions of each G20 nation to $PM_{2.5}$ concentration in those squares

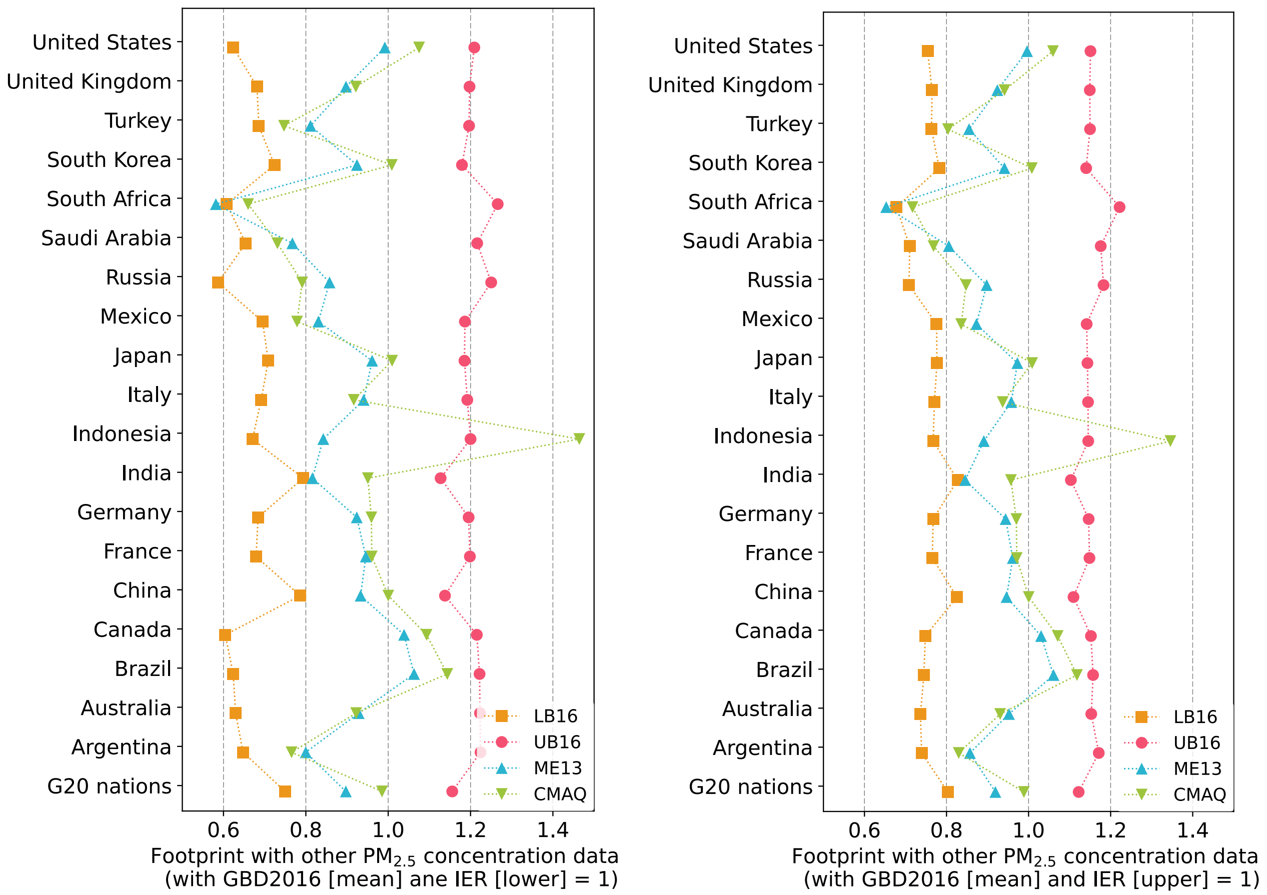

Supplementary Figure 12: Change in number of premature deaths footprint when other $PM_{2.5}$ concentration data (LB16, UB16, ME13, CMAQ) are used for grid concentration to estimate total premature deaths in each grid square, compared with 1 for premature deaths with IER (Integrated Exposure-Response) model (lower (left figure) and upper (right figure) value in 95% CI) and $PM_{2.5}$ concentration (mean) in GBD2016 (Global Burden of Disease 2016). LB16 and UB16 are the cases with lower and upper bound concentration within 95% confidence intervals (CI) of GBD2016, respectively. ME13 is the case with concentration (mean) in 2010 of GBD2013 and CMAQ the case with concentration calculated by the CMAQ (Community Multiscale Air Quality) modelling system.

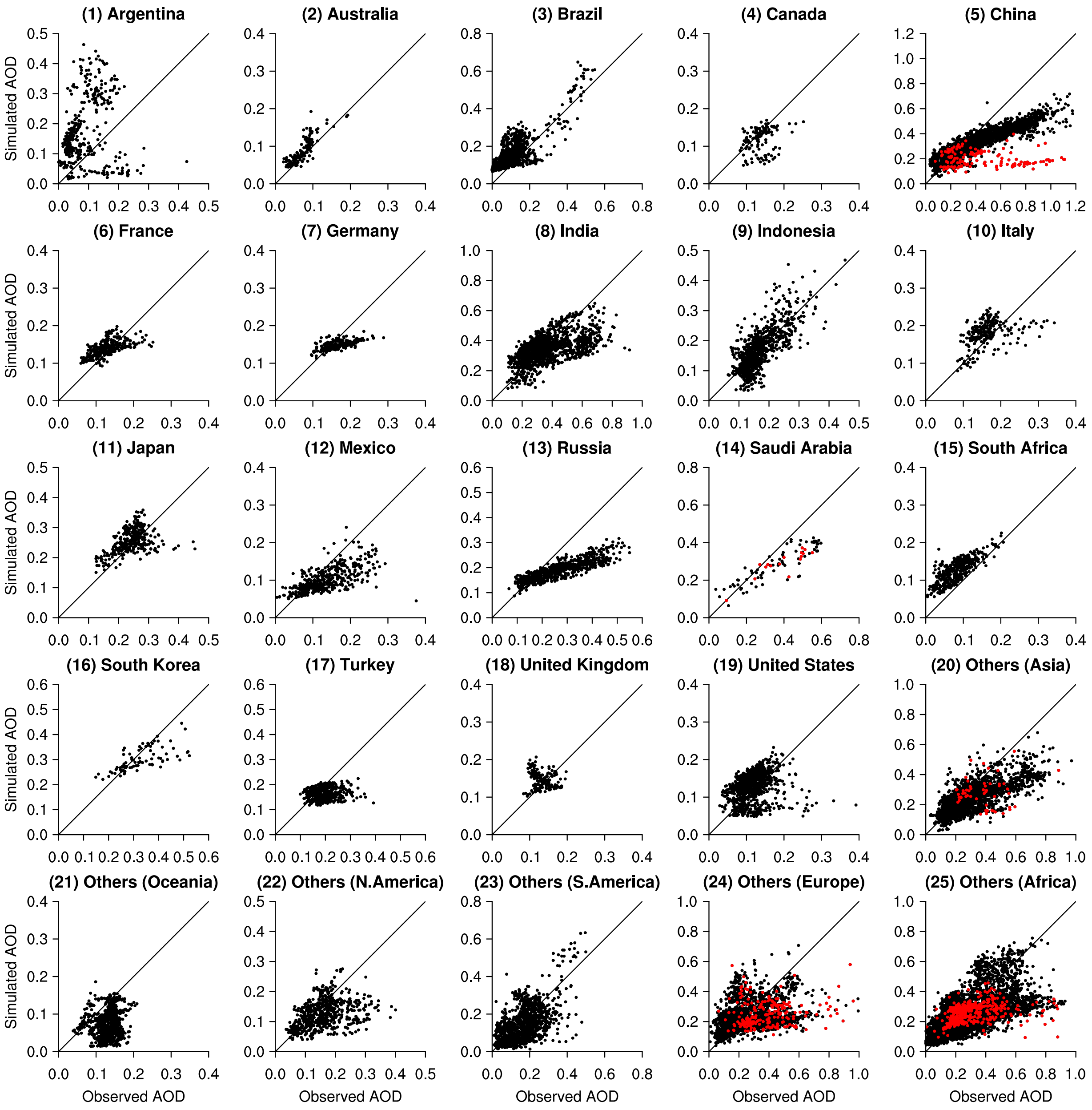

Supplementary Figure 13: Comparison of CMAQ-simulated AOD (Aerosol Optical Depth) with observed AOD; red dots are grid squares where dust concentration is indicated in GBD2013; grid squares with population density over 22.5 persons/km^2 are shown

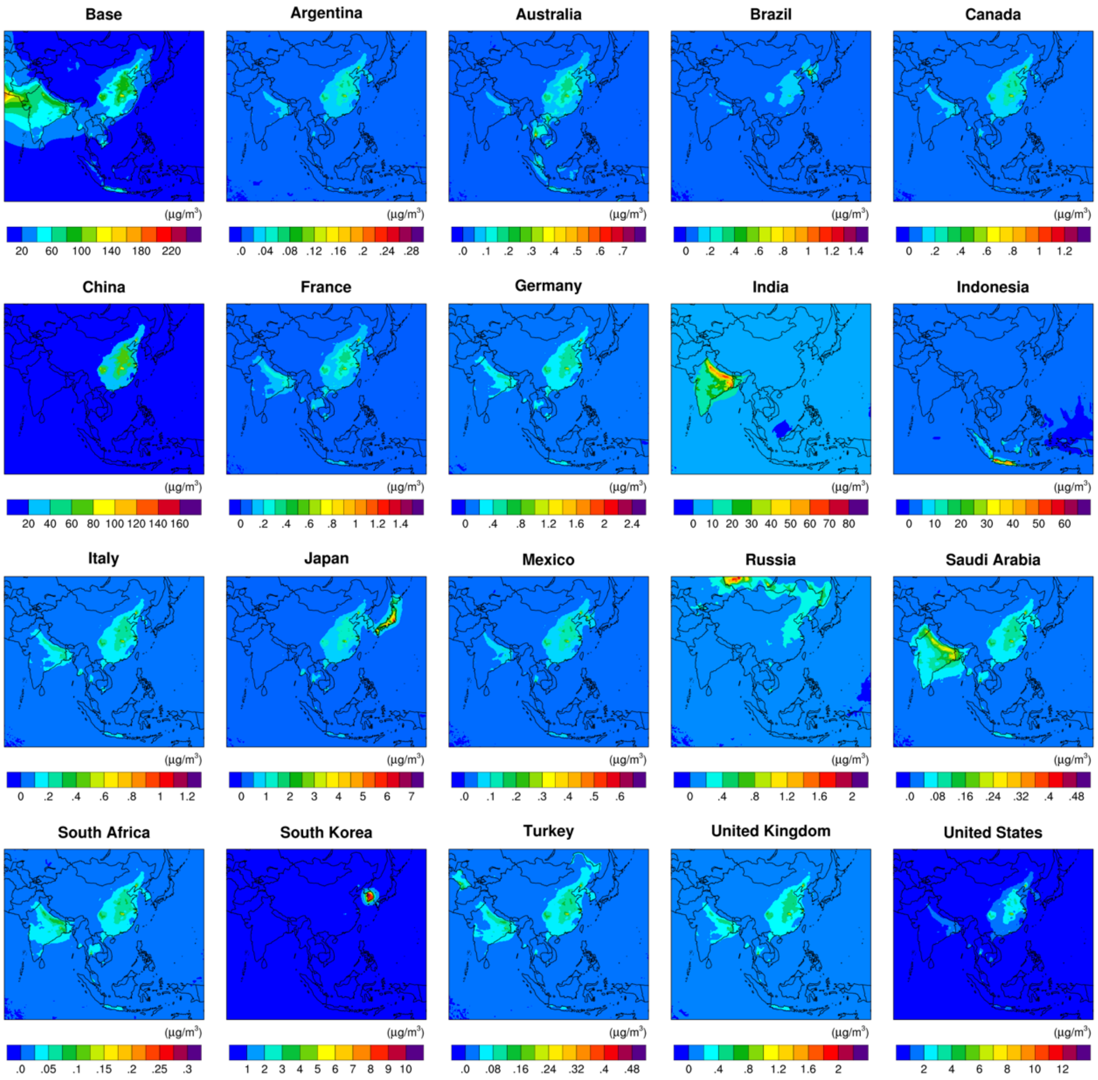

Supplementary Figure 14: Horizontal distribution of difference between mean annual $PM_{2.5}$ concentrations in base case and annual mean $PM_{2.5}$ concentrations due to consumption-based emissions of each G20 nation in Asian region

The maps were obtained from the NCAR Command Language (Version 6.6.2) [Software]. (2019). Boulder, Colorado: UCAR/NCAR/CISL/TDD. http://dx.doi.org/10.5065/D6WD3XH5

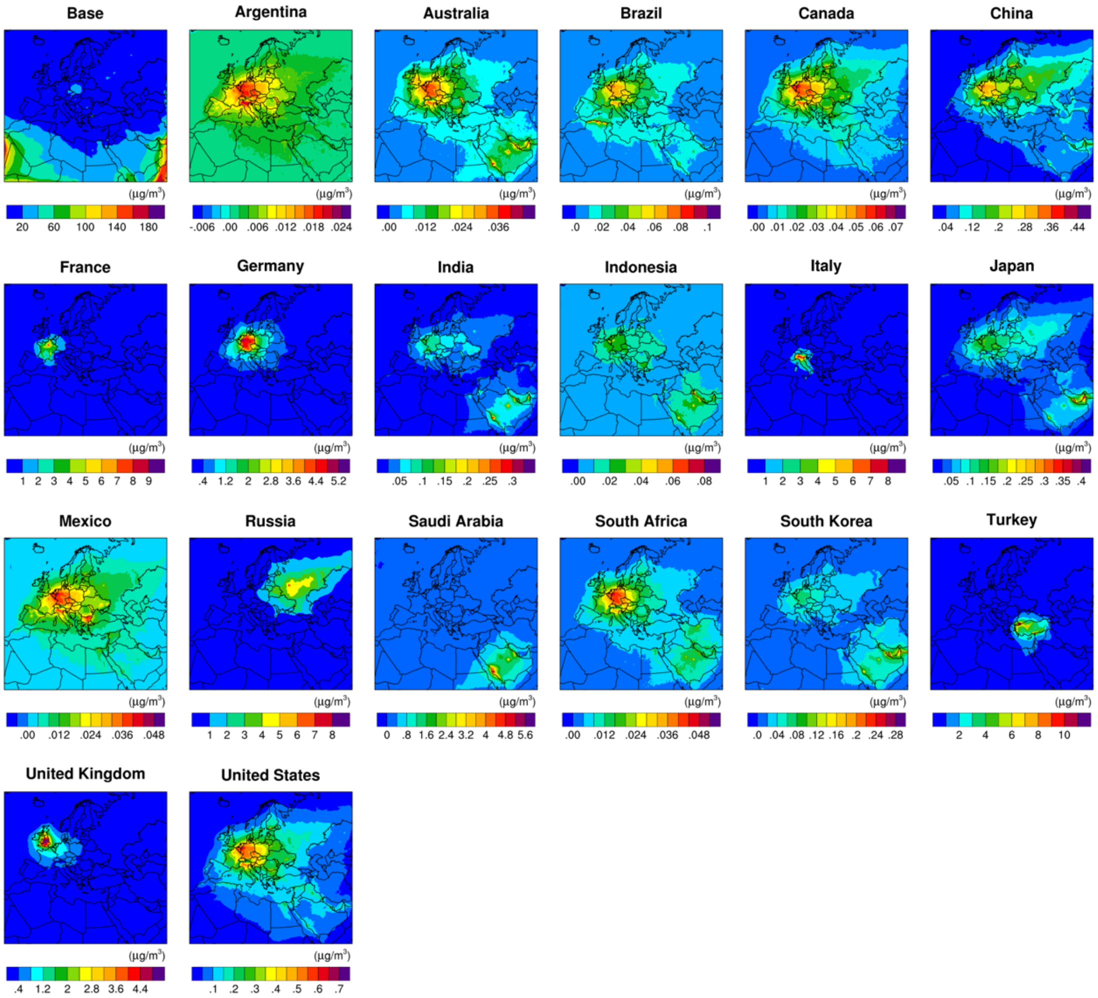

Supplementary Figure 15: Horizontal distribution of difference between mean annual $PM_{2.5}$ concentrations in base case and annual mean $PM_{2.5}$ concentrations due to consumption-based emissions of each G20 nation in European region

The maps were obtained from the NCAR Command Language (Version 6.6.2) [Software]. (2019). Boulder, Colorado: UCAR/NCAR/CISL/TDD. http://dx.doi.org/10.5065/D6WD3XH5

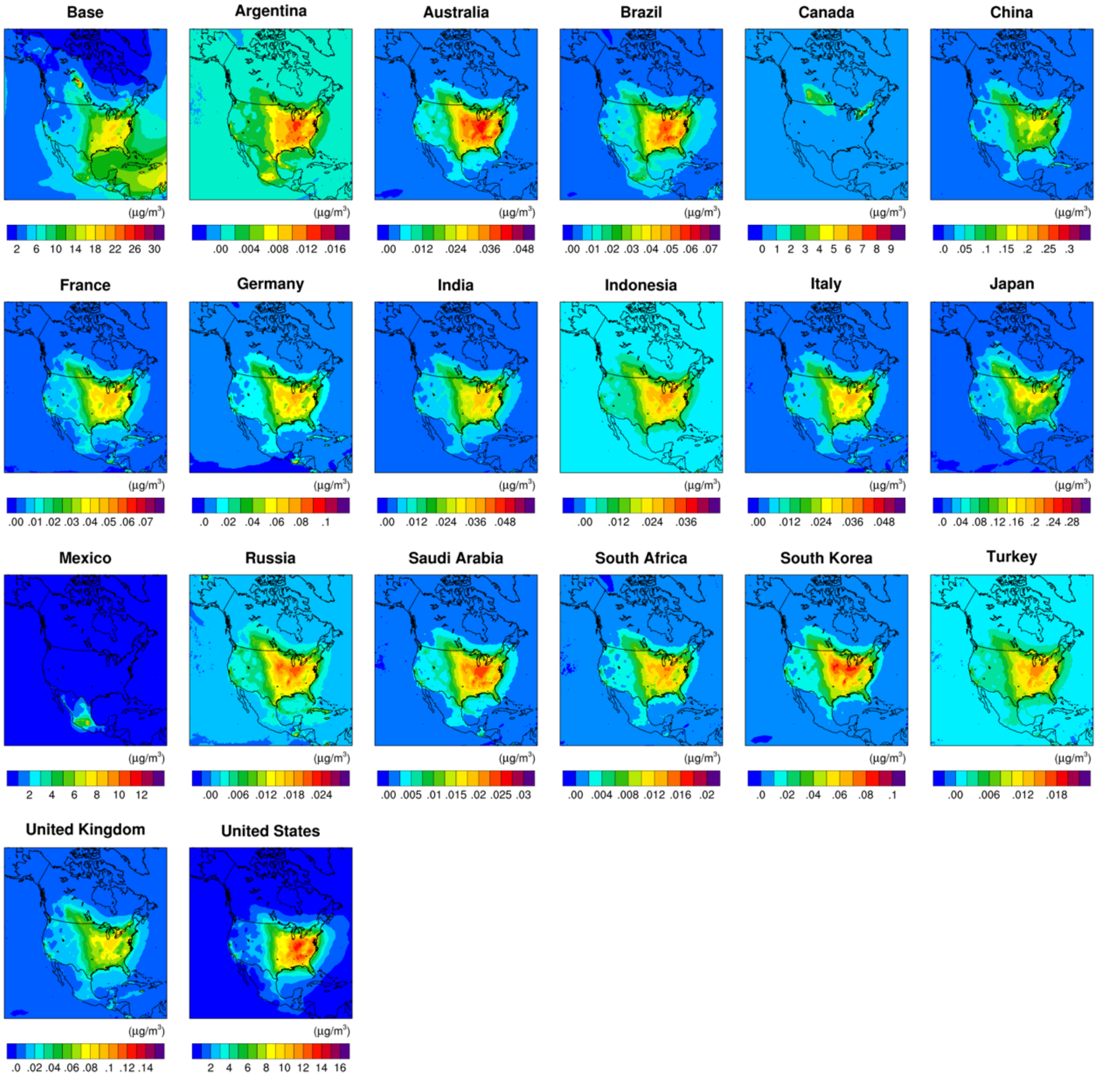

Supplementary Figure 16: Horizontal distribution of difference between mean annual $PM_{2.5}$ concentrations in base case and annual mean $PM_{2.5}$ concentrations due to consumption-based emissions of each G20 nation in North American region

The maps were obtained from the NCAR Command Language (Version 6.6.2) [Software]. (2019). Boulder, Colorado: UCAR/NCAR/CISL/TDD. http://dx.doi.org/10.5065/D6WD3XH5

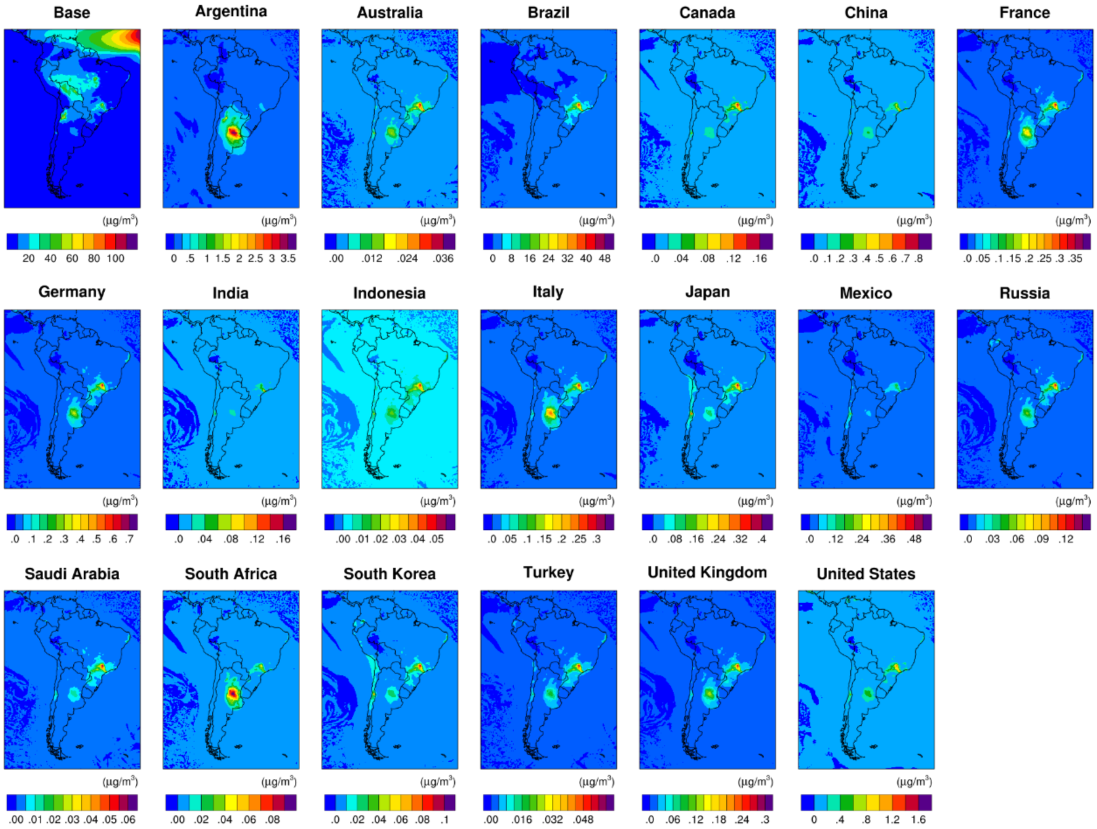

Supplementary Figure 17: Horizontal distribution of difference between mean annual $PM_{2.5}$ concentrations in base case and annual mean $PM_{2.5}$ concentrations due to consumption-based emissions of each G20 nation in South American region

The maps were obtained from the NCAR Command Language (Version 6.6.2) [Software]. (2019). Boulder, Colorado: UCAR/NCAR/CISL/TDD. http://dx.doi.org/10.5065/D6WD3XH5

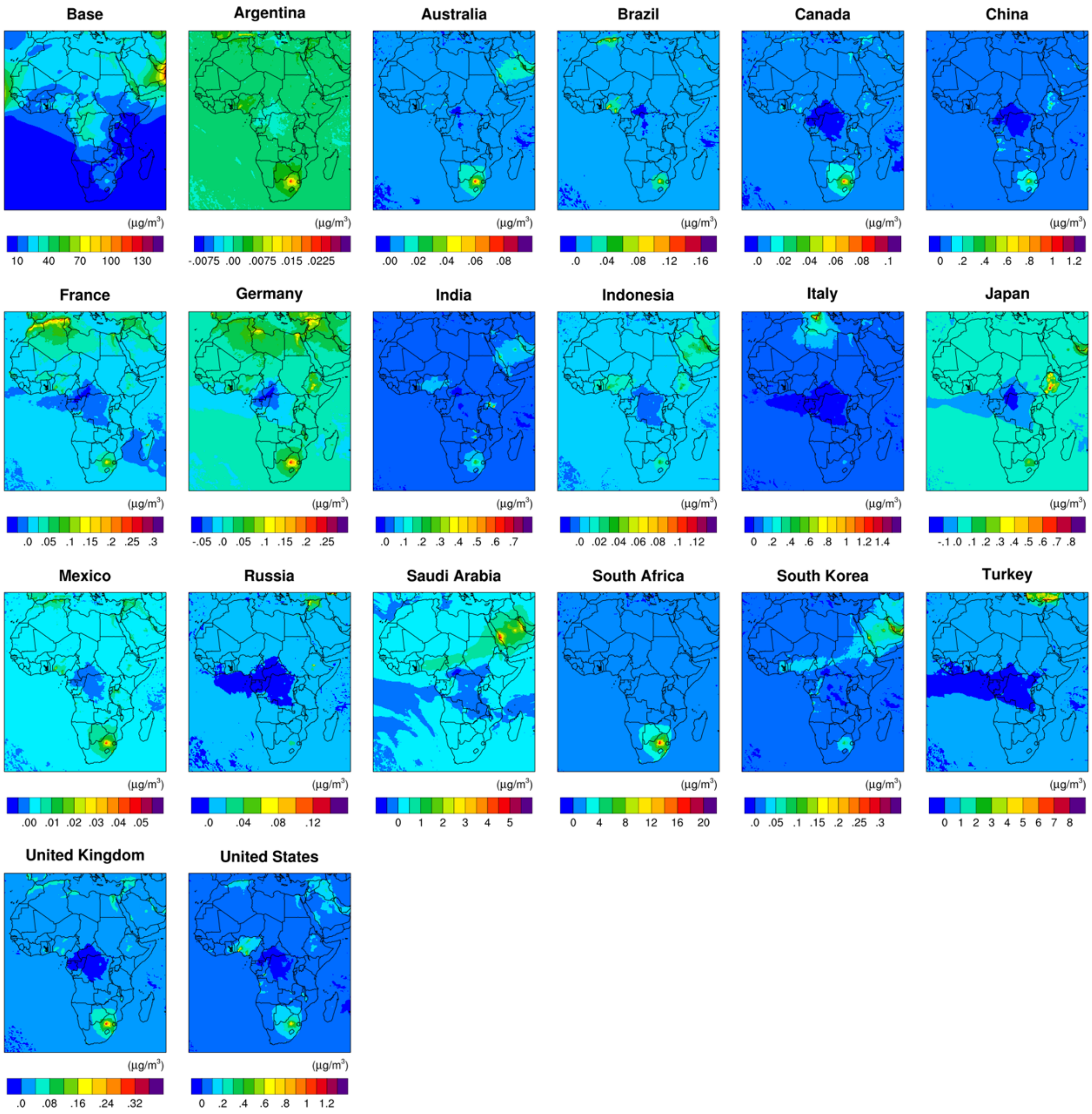

Supplementary Figure 18: Horizontal distribution of difference between mean annual $PM_{2.5}$ concentrations in base case and annual mean $PM_{2.5}$ concentrations due to consumption-based emissions of each G20 nation in African region

The maps were obtained from the NCAR Command Language (Version 6.6.2) [Software]. (2019). Boulder, Colorado: UCAR/NCAR/CISL/TDD. http://dx.doi.org/10.5065/D6WD3XH5

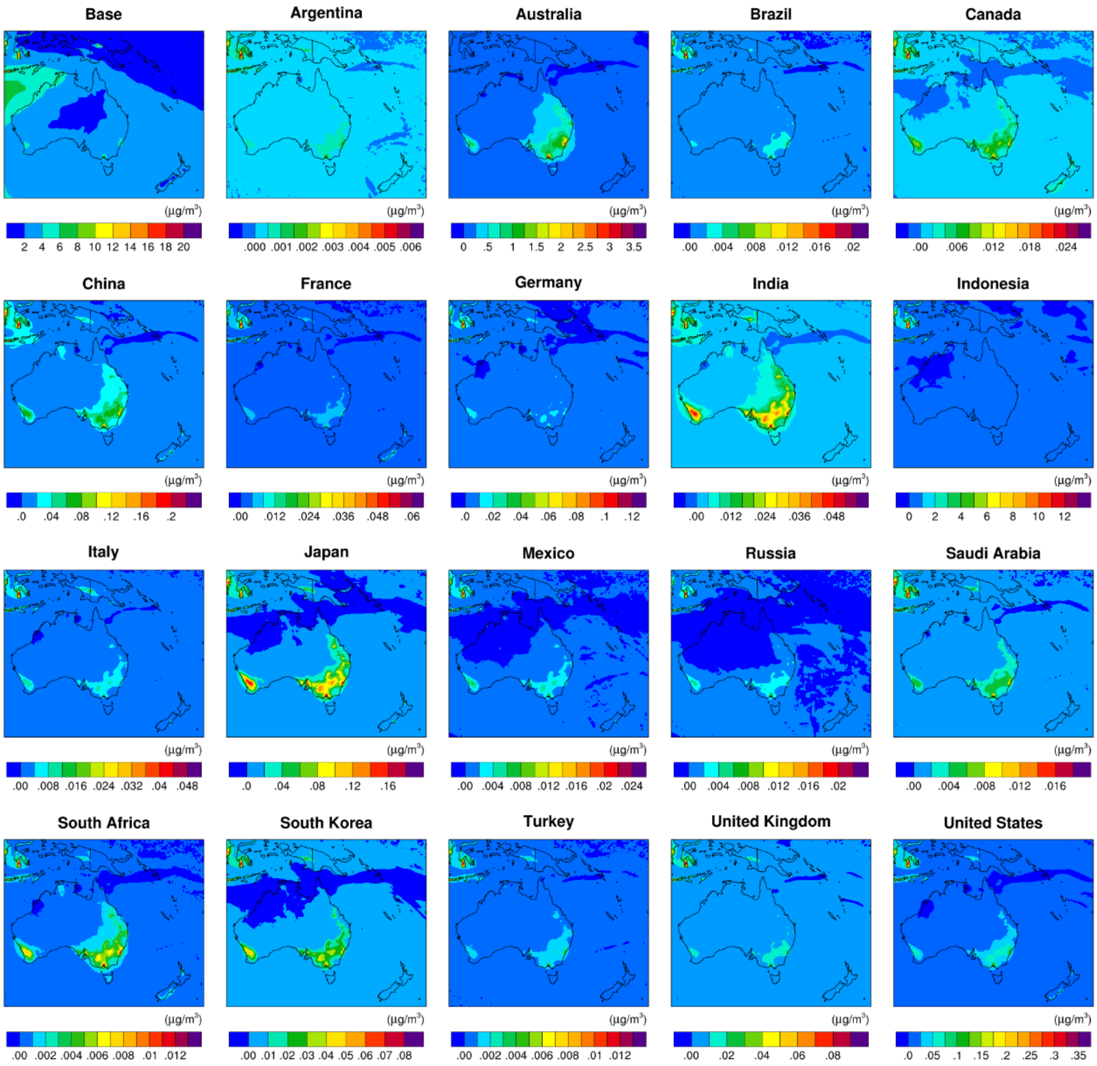

Supplementary Figure 19: Horizontal distribution of difference between mean annual $PM_{2.5}$ concentrations in base case and annual mean $PM_{2.5}$ concentrations due to consumption-based emissions of each G20 nation in Oceania region

The maps were obtained from the NCAR Command Language (Version 6.6.2) [Software]. (2019). Boulder, Colorado: UCAR/NCAR/CISL/TDD. http://dx.doi.org/10.5065/D6WD3XH5

Integrated exposure-response (IER) model

Our research quantified the health impacts of $PM_{2.5}$ by taking the number of premature deaths as an indicator. Five diseases were studied: ischemic heart disease (IHD), chronic obstructive pulmonary disease (COPD), stroke and lung cancer (LC), all of which have been linked to air pollution in previous studies[1-6], and lower respiratory infection (LRI), which kills more people worldwide than lung cancer[7]. First, the total number of premature deaths due to $PM_{2.5}$ was calculated per grid square using the method of Apte et al.[8], shown in Equation (1). The premature deaths associated with the consumption of each G20 nation were then determined by distributing this value according to the concentrations generated by each country's consumption-based emissions.

$$M_{i,j} = P_i \times \hat{I}_{j,k} \times \left(RR_j(C_i) - 1\right) \quad (1)$$

Here, $M_{i,j}$ is the number of premature deaths (persons) related to end point j in grid square i. P_i is population (persons) and $\hat{I}_{j,k}$ is the hypothetical underlying incidence (deaths/persons) in region k, calculated using Equations (2) and (3). $RR_j(C_i)$ is the relative risk (RR) corresponding to $PM_{2.5}$ concentration (C_i). $\hat{I}_{j,k}$ excludes the influence of $PM_{2.5}$ from the underlying mortality rate $I_{j,k}$, obtained from statistics. This method was also used by Wang et al. [3].

$$\hat{I}_{j,k} = I_{j,k} / \overline{RR}_{j,k} \quad (2)$$

$$\overline{RR}_{j,k} = \sum_{i=1}^{N} P_i \times RR_j(C_i) / \sum_{i=1}^{N} P_i \quad (3)$$

RR is an evaluative indicator of the strength of the association between disease and the causal factor of exposure. Assuming that exposure below the $PM_{2.5}$ reference concentration has a mortality risk of 1, RR indicates the relative size of the mortality risk arising from exposure to higher concentrations. This was calculated using the integrated exposure–response (IER) model of Burnett et al.[9] shown in Equations (4) and (5).

$$RR_j(C_i) = 1 + \alpha_j\left[1 - exp\left(-\gamma_j (C_i - C_0)^{\delta_j}\right)\right] \text{ for } C_i > C_0 \quad (4)$$

$$RR = 1 \text{ for } C_i \leq C_0 \quad (5)$$

Here, α_j, γ_j and δ_j are coefficients corresponding to end point j. To set these coefficients, RR was calculated at 0.1 μg/m^3 increments within a C_i range of 1－400 μg/m^3 using 1000 sets of parameter estimates (α_j, γ_j,δ_j,C_0) computed by Monte Carlo simulation to determine the range of uncertainty in the predictions of Burnett et al.'s model[9]. The 50%, 2.5% and 97.5% RR values were then calculated using multiple RRs corresponding to the C_i obtained above. Counterfactual concentration level C_0 was determined by reading the $PM_{2.5}$ concentrations from the graph, where RR starts at ≥1. Parameters α_j , γ_j and δ_j corresponding to the 50%, 2.5% and 97.5% values, respectively, were each determined using the non-linear least-squares method. With regard to end point j, parameter estimates for

stroke and IHD were made in 5-year age groups from ≥25 years to <80 years; estimates were also made in the ≥80 years group. Age-dependent parameters were not used for the other diseases (see Supplementary Table 2, Supplementary Figure 20). For this research, we adopted the parameter value corresponding to 50% for computing RR.

For underlying mortality rate $I_{j,k}$, the 2010 values in the GBD Results Tool listed in the Global Health Data Exchange (GHDx)[10] were used. Data on the five diseases were classified by country/region, gender and age group (5-year age groups from 0 to <80 years, and ≥80 years). For population P_i, LandScan[11] was used, which organizes population by gender and in 5-year age groups over 1 km grid squares.

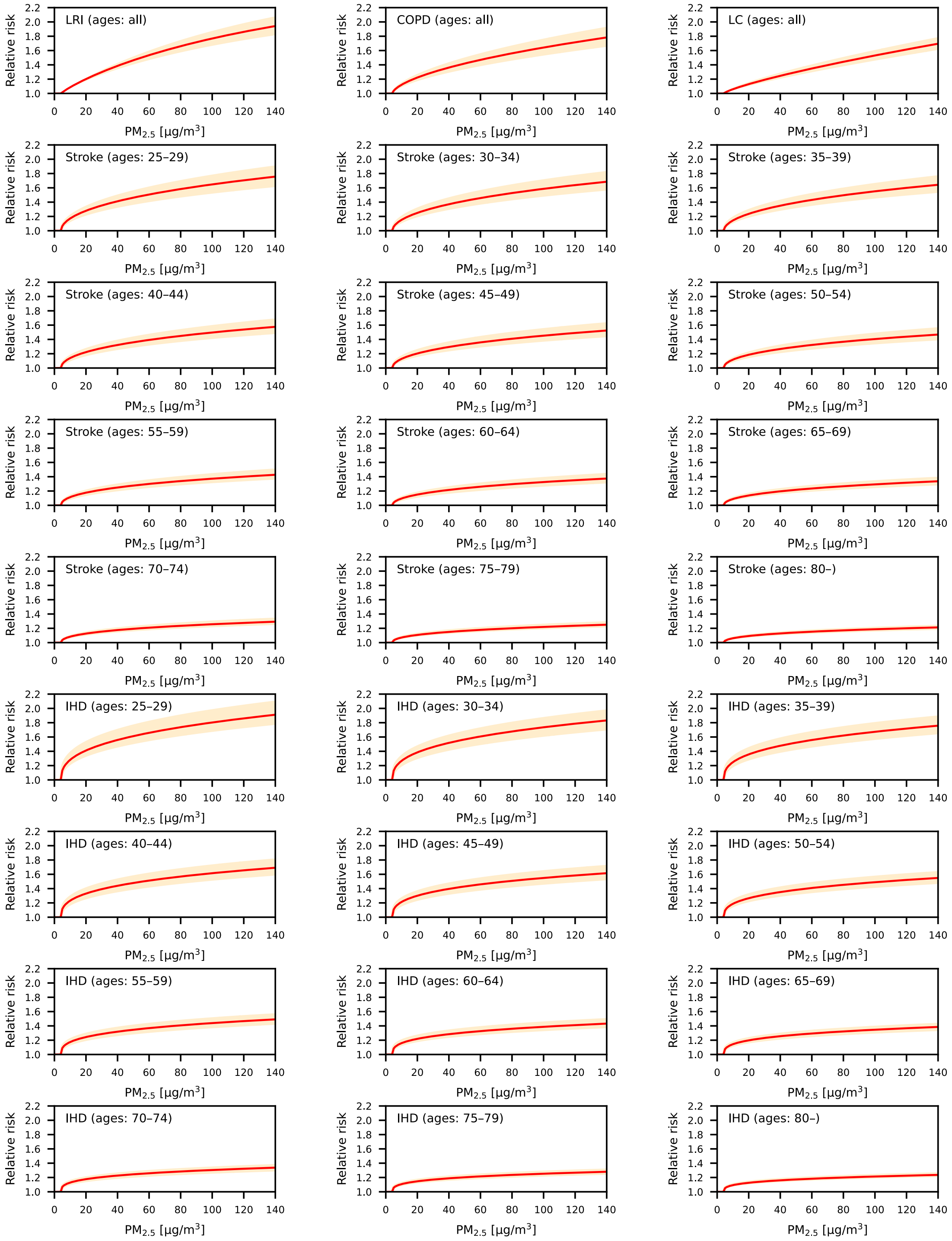

Supplementary Figure 20: Relationship between relative risk and $PM_{2.5}$ concentration defined by integrated exposure–response functions (IER); red lines represent IER based on 50th percentile of data samples, orange shaded areas IER based on 2.5th and 97.5th percentiles of samples, respectively.

References

1 Jiang, X. J. *et al.* Revealing the Hidden Health Costs Embodied in Chinese Exports. *Environmental Science & Technology* **49**, 4381-4388, doi:10.1021/es506121s (2015).

2 Xia, Y. *et al.* Assessment of socioeconomic costs to China's air pollution. *Atmos Environ* **139**, 147-156, doi:10.1016/j.atmosenv.2016.05.036 (2016).

3 Wang, H. K. *et al.* Trade-driven relocation of air pollution and health impacts in China. *Nat Commun* **8**, doi:ARTN 738
10.1038/s41467-017-00918-5 (2017).

4 Zhao, H. Y. *et al.* Effects of atmospheric transport and trade on air pollution mortality in China. *Atmos Chem Phys* **17**, 10367-10381, doi:10.5194/acp-17-10367-2017 (2017).

5 Zhang, Q. *et al.* Transboundary health impacts of transported global air pollution and international trade. *Nature* **543**, 705-709, doi:10.1038/nature21712 (2017).

6 Xia, Y., Guan, D., Meng, J., Li, Y. & Shan, Y. Assessment of the pollution‐health‐economics nexus in China. *Atmos Chem Phys* **18**, 14433-14443, doi:10.5194/acp-18-14433-2018 (2018).

7 Cohen, A. J. *et al.* Estimates and 25-year trends of the global burden of disease attributable to ambient air pollution: an analysis of data from the Global Burden of Diseases Study 2015. *The Lancet* **389**, 1907-1918, doi:10.1016/s0140-6736(17)30505-6 (2017).

8 Apte, J. S., Marshall, J. D., Cohen, A. J. & Brauer, M. Addressing Global Mortality from Ambient PM2.5. *Environmental Science & Technology* **49**, 8057-8066, doi:10.1021/acs.est.5b01236 (2015).

9 Burnett, R. T. *et al.* An Integrated Risk Function for Estimating the Global Burden of Disease Attributable to Ambient Fine Particulate Matter Exposure. *Environ Health Persp* **122**, 397-403, doi:10.1289/ehp.1307049 (2014).

10 IHME. *Global Health Data Exchange; GBD Results Tool*, <http://ghdx.healthdata.org/gbd-results-tool> (2018).

11 Center for International Earth Science Information Network - CIESIN - Columbia University. *Gridded Population of the World, Version 4 (GPWv4): Basic Demographic Characteristics, Revision 11. Palisades*, <https://landscan.ornl.gov/> (2018).
